# Machine Learning Classification of Migraine Using fNIRS During a Postural Task

**DOI:** 10.3390/brainsci16090927

**Published:** 2026-08-31

**Authors:** Emre Yorgancigil, Gülnaz Yükselen, Roksi Franci, Erkan Acar, Elif Ilgaz Aydinlar, Pinar Yalinay Dikmen, Ugur Uygunoglu, Aksel Siva, Abdullah Arcan, Feride Irem Simsek, Sinem Burcu Erdogan, Ata Akin

**Affiliations:** 1 Department of Biomedical Engineering, Graduate School of Natural and Applied Sciences, Acibadem Mehmet Ali Aydinlar University, Istanbul 34638, Türkiye; 2 Department of Biomedical Engineering, Faculty of Engineering and Natural Sciences, Acibadem Mehmet Ali Aydinlar University, Istanbul 34638, Türkiye; 3 Department of Neurology, School of Medicine, Acibadem Mehmet Ali Aydinlar University, Istanbul 34638, Türkiye; 4 Department of Neurology, School of Medicine, Istanbul University Cerrahpaşa, Istanbul 34320, Türkiye

**Keywords:** fNIRS, cerebrovascular reactivity, biomarker research, machine learning, migraine

## Abstract

Background: Migraine diagnosis and severity staging rest on clinical interviews according to the ICHD-3 criteria, with no validated objective biomarker. Functional near-infrared spectroscopy (fNIRS) is a portable and non-invasive method, but existing fNIRS migraine classification studies remain relatively small and rely on cognitive tasks. We assessed whether prefrontal hemodynamic responses with a head-down-to-knees maneuver separate migraine patients from controls, and high- from low-severity migraine. Methods: Prefrontal fNIRS, including short separation channels, was recorded during the maneuver in 50 interictal migraine patients and 52 controls screened for the absence of migraine, serious chronic conditions and hypertension. Nine hemodynamic parameters per chromophore (HbO, Hb and HbT) across thirty channels entered a hypothesis-neutral pipeline of 270 candidate pipelines (3 chromophores × 3 feature selection strategies × 30 classifiers) with no predefined region of interest. Results: Deoxyhemoglobin features selected by embedded L1 regularization with shrinkage-regularized linear discriminant analysis separated the groups with 92% balanced accuracy on the internal hold-out (92% sensitivity, 92% specificity, ROC-AUC 0.99; permutation *p* = 3 × 10^−4^), against 87% in development-set cross-validation and 88% under nested selection cross-validation, with a selection bias of +0.03. High- vs. low-severity classification (19 high, 31 low) did not exceed chance under nested validation (45%; permutation *p* = 0.45). Conclusions: A wide-scale pipeline achieved a robust, validated separation of migraine from screened controls, carried by a distributed venous-weighted deoxyhemoglobin signature; specificity against other headache disorders remains to be established. Attack frequency severity was not separable above chance, indicating that scalar hemodynamic descriptors are sufficient for a categorical but not a graded contrast.

## 1. Introduction

Migraine is a highly prevalent and disabling neurological disorder, ranking among one of the leading global causes of years lived with disability and imposing a substantial burden on patients and healthcare systems worldwide. Its estimated global prevalence is approximately 14%, and it is nearly three times more common in women than in men [1,2]. Clinically, the severity of migraine is stratified by monthly headache day frequency. Episodic migraine (EM) is defined as fewer than 15 headache days per month and subdivided into low-frequency (<8 days/month) and high-frequency (8–14 days/month) groups. Chronic migraine (CM) is defined as a headache frequency of 15 or more days/month for >3 months of duration [3,4]. Accurate frequency-based severity classification is clinically important for guiding therapeutic strategy, such as whether only acute treatment is sufficient or preventive therapy should be added. Effective prophylaxis in higher-frequency migraine patients can both improve attack control and prevent the disease’s progression from episodic to chronic migraine, a process primarily driven by modifiable factors such as acute medication overuse [2,4].

Both the differential diagnosis of migraine from other headaches and its frequency-based severity classification (episodic vs. chronic) still almost entirely depend on clinical interviews according to ICHD-3 criteria. To date, no biochemical, cerebrospinal fluid, or neuroimaging marker has achieved broad acceptance for validating a migraine diagnosis [5,6,7]. This gap makes the search for an objective neuroimaging or electrophysiological biomarker a valid and active research question, and a growing body of neuroimaging studies employ machine learning (ML) classification algorithms to discriminate migraine patients from controls or to separate migraine subtypes. Structural MRI approaches have used both deep learning on raw brain images and classical cortical morphometry features (thickness, surface area, volume, and curvature) to separate migraine patients from controls, reporting accuracies ranging from roughly 75% up to about 97% for migraine with aura (MwA) cohorts [8,9]. Resting-state fMRI studies have mainly focused on functional connectivity features (static, dynamic, or both) and have achieved over 90% accuracy in distinguishing migraine from controls. Distinct connectivity patterns in the precuneus, cuneus, and frontoparietal networks were also found [10,11,12]. EEG-based classifiers have used resting-state functional connectivity or spectral and time frequency representations of the electrophysiologic signal. Reported accuracies varied from the 80% band upward, and occipital power changes around 12 Hz have shown the most robust, cohort-validated signal [13,14,15,16].

ML classification studies with an fNIRS methodology are very limited. Chen et al. [17] recorded prefrontal oxyhemoglobin (HbO) and deoxyhemoglobin (Hb) chromophores with a four-channel wearable system during a mental arithmetic stress task in 34 participants (13 healthy, 9 CM, and 12 patients with medication overuse headache (MOH)). LDA/QDA estimators reached 90% test accuracy for healthy vs. migraine classification and for separating the CM and MOH subgroups (sensitivity/specificity of 100%/75% and 75%/100%, respectively) under leave-one-out cross-validation. Kara Gulay et al. [18] recorded 16-channel prefrontal HbO and Hb during a Victoria Stroop test in 32 participants (8 patients with migraine with aura (MwA), 12 with migraine without aura (MwoA), and 12 healthy controls) and extracted time frequency domain features across the PFC. An XGBoost estimator on time frequency ΔHbO features from the left PFC achieved 92% balanced accuracy, 89% sensitivity, and 95% specificity in classifying the MwA, MwoA, and healthy control subgroups. 

In the present study, we evaluated the hemodynamic effects of a head-down-to-knees maneuver in healthy controls and migraine patients during fNIRS recording. Functional near-infrared spectroscopy (fNIRS) is a non-invasive, comfortable, and practical neuroimaging method that enables continuous data acquisition in naturalistic settings [19], and it has been applied across a range of neurological and clinical conditions, including migraine [20,21,22,23]. Postural tasks of the head have a direct and quantifiable influence on cerebral hemodynamics. Because the cerebral circulation sits on a hydrostatic column, changes in head position alter perfusion pressure at the level of the circle of Willis. Invasive intracranial pressure measurements in humans show that intracranial pressure is lowered in the upright seated posture relative to the supine position, establishing body and head position as a major factor for intracranial pressure [24]. Head maneuvers affect cerebral perfusion pressure more than metabolic demand and therefore engage myogenic and autoregulatory mechanisms rapidly, challenging vaso-mechanical and cerebrovascular regulation [25]. Prefrontal HbO, Hb, and tissue oxygenation have been recorded during 90° head-up and −6° head-down tilt in healthy adults [26], and prefrontal fNIRS recorded across five discrete phases of a head-up tilt-table test distinguished healthy controls from patients with electrical burns, in whom reduced cerebral perfusion and decreased HbO were observed [27]. Direct application of postural head maneuvers to migraine remains limited; to our knowledge, the only prior fNIRS study of head-down maneuvers in this population is a small-scale investigation of 12 interictal migraineurs and 12 controls, which reported a decreased total hemoglobin response over the right hemisphere in the migraine group [28].

Considering the preceding studies, our study aimed to discriminate the cerebral hemodynamic profiles of patients with migraine from those of controls, and of high- from low-severity migraine subtypes, using machine learning classification of fNIRS signals recorded during a head-down-to-knees postural task. Two classification problems were addressed: migraine vs. control (50 patients, 52 controls; *n* = 102) and high- vs. low-severity migraine (19 high, 31 low; *n* = 50). High-frequency episodic and chronic migraine were grouped as the high-severity subtype because the 15-day boundary separating episodic from chronic migraine is a classificatory convention rather than a biological discontinuity, and patients with sustained high-frequency episodic migraine closely resemble those with chronic migraine in disability, symptom burden, and treatment requirements [29], while additionally carrying an elevated risk of frequency progression. Given the broad range of the model selection search relative to the available sample, both analyses are exploratory. Our objective was therefore to determine whether fNIRS-derived hemodynamic responses discriminate not only distinct diagnostic entities but also clinically meaningful levels of migraine burden.

Our study is distinguished by two main features. First, its cohort size is substantially larger than that of prior fNIRS ML studies on migraine, affording tighter and more reliable performance estimates. Second, rather than feeding minimally processed fNIRS signals directly to an estimator, we characterized each recording with a set of physiologically motivated hemodynamic parameters. We chose the hemodynamic area under the curve (hAUC), the peak and dip amplitudes (MAXPeak, MINDip, and MINRec), the signal range (RANGE), and the signal power (POWER) to sharpen the physiological meaning of the hemodynamic response rather than its raw waveform alone.

We tested our hypothesis that migraine patients can be discriminated from controls screened for the absence of migraine, serious chronic conditions, and hypertension, and that high- and low-severity migraine can be separated from one another by using a deliberately broad range of ML-based classification approaches. Our broad range of ML classification pipelines evaluates these hemodynamic parameters without pre-imposing a region of interest, a privileged chromophore, a single feature selection strategy, or a particular estimator family, and without shaping the pipeline around migraine-specific physiological expectations. Under this hypothesis-neutral classification pipeline design, any convergent signal such as a hemodynamic parameter, a chromophore, a prefrontal region, or an estimator model family can emerge as an empirical finding rather than a built-in assumption. As a secondary aim, we sought to identify which hemodynamic parameters and prefrontal regions contribute most to classification performance, which could serve as candidate biomarkers for migraine and its severity.

## 2. Materials and Methods

### 2.1. Participants

Fifty-two healthy control (HC) subjects (45 females and 7 males, mean age: 31.5 ± 13.3 years (SD)) and fifty migraine patients (MP, 45 females and 5 males, mean age: 34.6 ± 11.5 years (SD)) were enrolled in our study. Consecutive patients presenting to the tertiary headache outpatient clinics who fulfilled the eligibility criteria were invited to participate. Migraine diagnoses were established by neurologists according to the International Classification of Headache Disorders, 3rd edition (ICHD-3) [3]. The sample size of the study was determined with G*Power (version 3.1.9.7; Heinrich Heine University Düsseldorf, Düsseldorf, Germany). The effect size was derived from previously reported group differences between patients with migraine and controls in the latency of the HbO response, measured by fNIRS during a cerebrovascular reactivity challenge [20,23], corresponding to a Cohen’s f = 0.29. For a two-tailed comparison of two independent groups at α = 0.10 with statistical power of 0.90, the required sample was 52 participants per group, giving a target total of 104. All migraine participants were evaluated during the interictal period, and no recordings were performed during an acute migraine attack. Patients were grouped by the neurologists of the study group according to their attack frequency as LFEM (LM, low-frequency episodic migraine, *n* = 31), HFEM (*n* = 6) and CM (*n* = 13). For the classification task, high episodic (HFEM) and chronic migraine (CM) patients were grouped under the high-severity migraine class (HFEM + CM = HM, *n* = 19) (Table 1). All participants were native Turkish speakers and residents of Istanbul, where the recruiting tertiary headache outpatient clinics are also located. Inclusion criteria for the MP group were to have at least one migraine attack in the preceding month and being in an attack-free interictal period at the time of recording. For the HC group, inclusion criteria required the absence of any serious chronic condition, migraine, and hypertension. 

Each subject provided informed consent before enrolment. The study protocol was approved by the Clinical Research Ethics Committee of Acıbadem University, Istanbul, Türkiye (2023-11/71), and all procedures were conducted in accordance with the principles outlined in the most recent version of the Declaration of Helsinki. The data collection phase of the experiment was conducted between February and November 2025.

### 2.2. Experimental Protocol and fNIRS Data Acquisition

The experiment was conducted in a quiet, half-dark room. Participants sat comfortably approximately 70 cm from a 15-inch monitor. Prior to data collection, the experimental protocol was briefly explained to each participant. During the session, participants were asked to relax, limit unnecessary movement, and focus on the cues and instructions.

**Figure 1:**
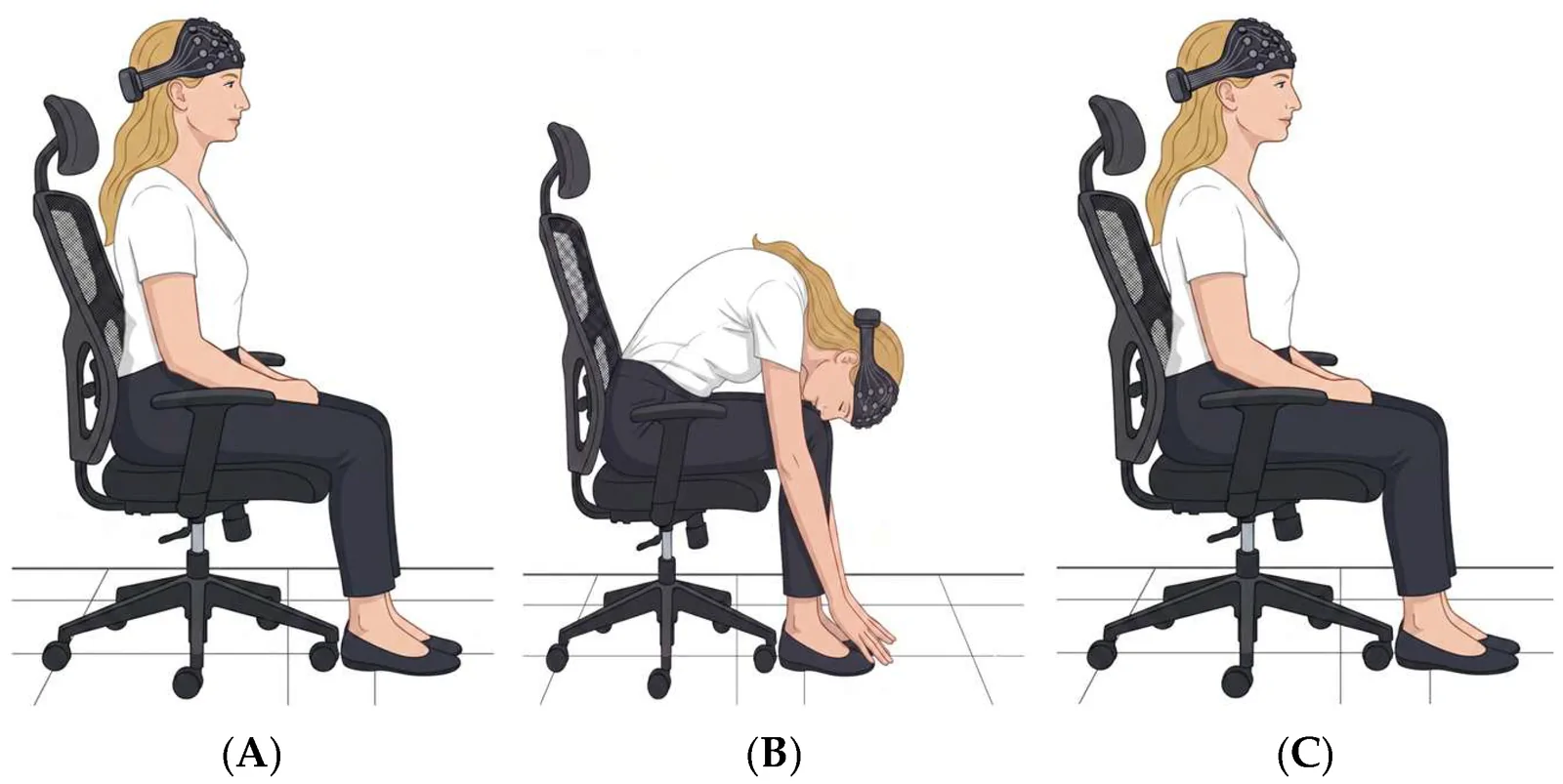
Experiment design: upright position (**A**), head down to knees (**B**), and back to upright (**C**).

The experimental protocol began with a 40 s hemodynamic baseline recording. A single postural task session consisted of a 90° head-down-to-knees maneuver, maintaining the position for 20 s, and then returning to an upright position. The head-down-to-knees maneuver was performed in two versions, in which participants followed verbal instructions from the experimenter: fast head down with fast return to upright, and slow head down with slow return to upright. A 30 s rest period was applied after each fast and slow session. Head-down-to-knees task sessions were repeated three times in random order (Figure 1).

Cortical hemodynamic activity was recorded with an fNIRS neuroimaging device (NIRSport, NIRx Medical Technologies, Berlin, Germany). A probe comprising 22 long and 8 short separation channels was positioned over the PFC and premotor cortex regions. The fNIRS probe was equipped with eight light-emitting sources (760 and 850 nm) and eight detectors. Source–detector pairs separated by 3 cm were defined as channels. Raw optical signals were acquired at a sampling rate of 7.81 Hz.

### 2.3. Data Preprocessing and Feature Extraction

In the first step, raw optical signals were transformed into optical density (OD) changes. The OD data were then converted into concentration changes of oxyhemoglobin (HbO) and deoxyhemoglobin (Hb) chromophores with the modified Beer–Lambert law [30], as already implemented in Homer2 scripts. Probe sensitivity profiles were computed with the AtlasViewer toolbox of the Homer2 (Athinoula A. Martinos Center for Biomedical Imaging, Massachusetts General Hospital, Charlestown, MA, USA) software to confirm that every channel collected data from the first few millimeters of the frontal cortex [31,32,33]. The optical sensitivity profile of each channel was first registered to a standard brain template using the 10/5 global EEG electrode system. Channel locations were additionally mapped onto a standard brain in MNI space with the NIRS_SPM toolbox [34,35]. The Brodmann areas that each channel’s photons passed through were identified using the spatial registration toolbox of NIRS_SPM with Rorden’s brain atlas [36]. The specific Brodmann areas covered by each channel of the currently used forehead probe were reported in an earlier study with the same device and settings [33]. All further signal processing and analysis were carried out with custom MATLAB 2022b scripts (MathWorks, Natick, MA, USA).

All fNIRS signals were preprocessed prior to analysis to reduce noise, correct artifacts, and ensure consistency across participants. The preprocessing pipeline consisted of channel quality screening, wavelet-based denoising, and filtering and downsampling steps. Channels containing missing (NaN) values were identified separately for HbO and Hb signals and replaced with the mean of the remaining valid channels within the same separation class as long or short channels. Channel replacement was required for the 11 HC participants, with a 7.5% replacement rate (total replaced channels/22 long channels × n participants); for the 13 MP participants, a 14.3% replacement rate. No channels were replaced for the remaining participants. Then, a coefficient of variation (CV) threshold was applied to the preprocessed signals. Channels exceeding a CV threshold of 0.2 were identified as low quality and replaced with the mean signal of the remaining valid channels within the same hemispheric group (left or right). Prior to wavelet-based denoising, linear detrending was applied to each channel to remove slow baseline drifts from the hemodynamic signals. To attenuate motion-related and transient artifacts, discrete wavelet decomposition was applied to the signals using a Daubechies 4 (db4) wavelet with a decomposition level of 6. The signal was reconstructed by retaining detail coefficients at decomposition levels 3 through 6 and the approximation coefficients at level 6, while discarding detail coefficients at levels 1 and 2, which correspond to high-frequency noise components unrelated to the hemodynamic response of interest. Wavelet-based methods have been widely used in fNIRS preprocessing for motion artifact reduction while maintaining physiological signal integrity [37].

**Figure 2:**
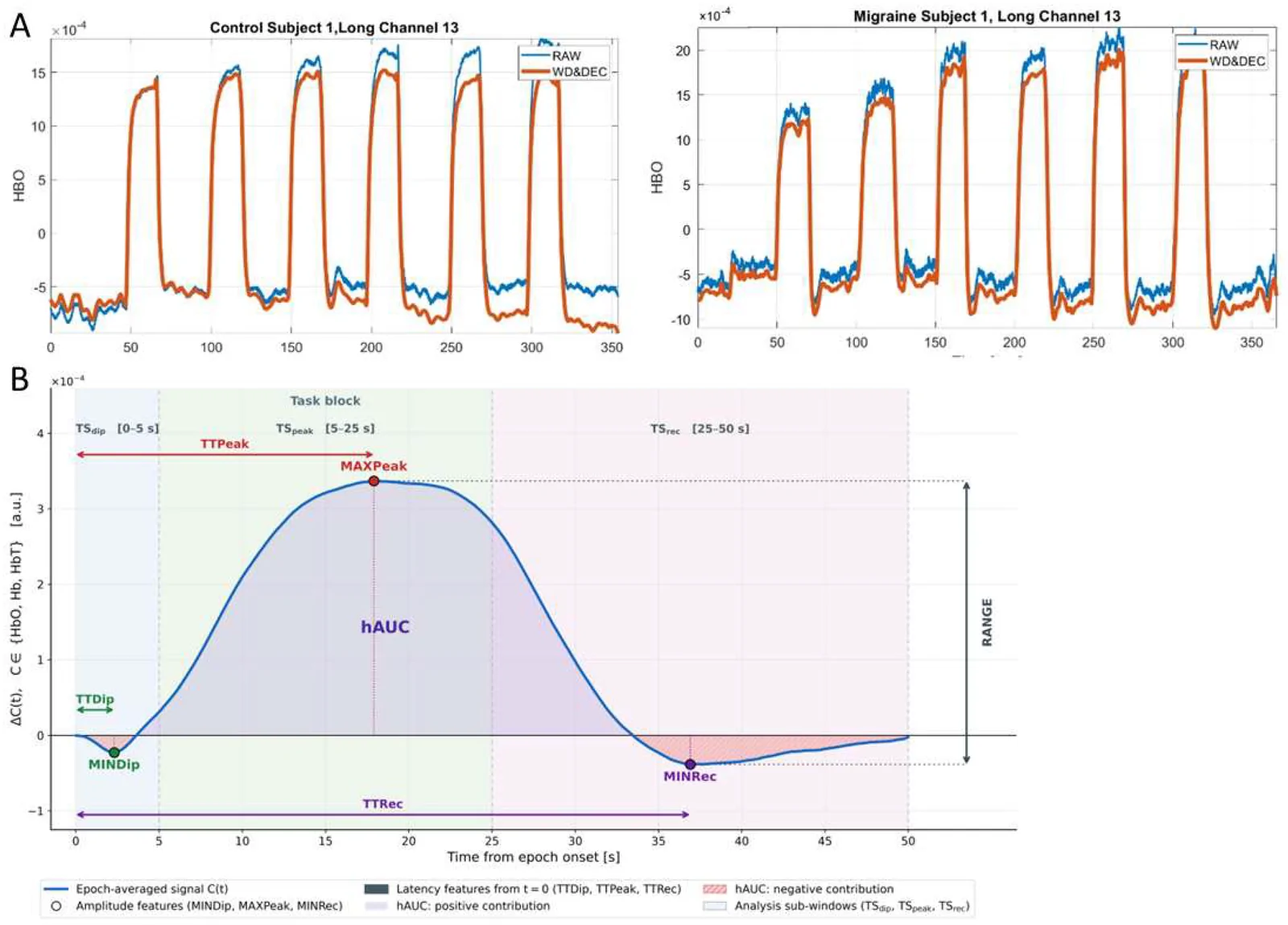
Representative HbO signals from selected channel (ch 13) before and after preprocessing. The comparison illustrates attenuation of transient fluctuations and high-frequency noise while preserving task-evoked hemodynamic response patterns in both control (HC) and migraine (MP) participants (**A**). Hemodynamic features extracted from each epoch-averaged fNIRS time course (Table 2). Representative epoch-averaged signal C(t), C ∈ {HbO, Hb, HbT}, in arbitrary units, time-locked to epoch onset (t = 0) and shown over the full epoch TS. Task block denotes the stimulation period (0–20 s). Filled circles mark the amplitude features MINDip, MAXPeak, and MINRec. Double-headed arrows beginning at t = 0 give the corresponding latencies TTDip, TTPeak, and TTRec. The shaded area is hAUC, the signed integral of C(t) over TS: samples above baseline (purple) contribute positively, samples below baseline (red hatching) negatively. RANGE (right-hand arrow, dotted guides) is the global peak-to-peak amplitude, max C(t) − min C(t) over TS, irrespective of which phase supplies either extremum. POWER, the mean squared amplitude of C(t) over TS, is a single scalar summarising the whole epoch and has no graphical locus on the trace. All features are computed identically for the three chromophores (**B**).

Following the wavelet-based denoising, signals were low-pass filtered using a fourth-order Butterworth filter with a cutoff frequency of 0.5 Hz to suppress high-frequency physiological oscillations. The filtered signals were subsequently downsampled by a factor of 4 (from 7.8125 Hz to approximately 1.95 Hz) to reduce computational load. Finally, the preprocessed data were segmented into epochs based on the onset of the head-down-to-knees task. Each epoch consisted of a 10 s pre-stimulus period, a 20 s task period, and a 30 s post-stimulus period. To isolate task-evoked changes and account for baseline shifts, each epoch was subsequently baseline corrected by subtracting the mean signal value calculated over the 10 s pre-stimulus period. After the segmentation, a set of hemodynamic parameters was calculated from each of the individual task epochs to characterize the hemodynamic response of the task (Table 2, Figure 2).

### 2.4. Classification Pipelines

Feature space and tasks: For each subject, 540 hemodynamic features per chromophore were extracted as 2 task conditions (slow/fast head down to knees) × 9 hemodynamic parameters (hAUC, MAXPeak, MINDip, MINRec, POWER, RANGE, TTDip, TTPeak, and TTRec) × 30 fNIRS channels, encoded uniformly as {Condition}_{Chromophore}_{Metric}_{Channel} (e.g., SlowTilt_Hb_MAXPeak_Ch17). Two classification tasks were defined: migraine patients vs. healthy controls (MP vs. HC, *n* = 102 (MP = 50, HC = 52)), high- vs. low-severity migraine (HM vs. LM, *n* = 50 (HM = 19, LM = 31)). Each task × chromophore × hemodynamic parameter combination was processed through an identical pipeline.

Pipeline architecture and leakage control: Every estimator model was wrapped in a single Python scikit-learn pipeline [38,39], so imputation, scaling, and feature selection were refit on the training fold only and applied unchanged to the hold-out fold. Missing values were imputed by the per feature median (SimpleImputer(strategy = “median”, keep_empty_features = True)), preserving channels whose metric was entirely undefined to maintain constant feature dimensionality across subjects. Features were z-score standardized (StandardScaler) to neutralize heterogeneous numerical scales across hemodynamic parameters. All steps downstream of imputation, including feature selection and final model fitting, were strictly fold encapsulated; no transformation was ever fit on test data.

Feature selection (FS) scenarios: Three FS strategies were compared at a fixed budget of k = 7 features (√n heuristic for 52 HC and 50 MP subjects separately). They provide complementary perspectives on statistical separability, non-redundant evidence, and linear model relevance.

1. Univariate filter (SelectKBest) ranking features by the ANOVA F-statistic against task labels.
2. Minimum redundancy maximum relevance (mRMR) in additive FCD form, with relevance defined as the normalized ANOVA F-score and redundancy as the mean absolute Pearson correlation with already-selected features, computed via a vectorized correlation matrix.
3. Embedded L1-regularized logistic regression (SelectFromModel with penalty = “l1”, solver=“saga”, C = 0.1, class_weight = “balanced”) retaining features with non-zero coefficients.

Classifier pool: Thirty estimators from nine classification algorithm families were employed: linear discriminants (logistic regression with L2, L1, elastic-net at two l1_ratio settings, Ridge, LinearSVC, and SGD with log-loss); classical discriminant analysis (standard LDA, shrinkage-LDA via LSQR with automatic Ledoit–Wolf regularization, and regularized QDA at reg_param = 10^−2^); kernel methods (SVM with RBF and polynomial-degree-2 kernels); instance-based learners (k-NN at k = 3 and k = 5); probabilistic models (Gaussian Naïve Bayes and Gaussian-process classifier with RBF kernel); a domain-adapted chemometric baseline (PLS-DA with 3 and 5 latent components, implemented via one-hot Y encoding and argmax decoding to support binary and multiclass settings); tree-based and boosted ensembles (Decision Tree, Random Forest, Extra Trees, Gradient Boosting, HistGradientBoosting, AdaBoost, XGBoost, LightGBM, and CatBoost); a shallow neural network (MLP with two hidden layers); and two meta-learners (a soft-vote ensemble and a stacking classifier with logistic regression as the meta-estimator). All models that expose a class_weight parameter were configured with class_weight = “balanced”, which weights samples inversely proportional to class frequency and directly addresses the 31:19 high- vs. low-migraine-severity imbalance in the dataset. Our estimator pool was implemented within a scikit-learn-style pipeline framework and was designed to provide broad coverage of major supervised classification paradigms commonly used in the open source Python machine learning ecosystem [38,39].

Validation: Before any analysis, the experimental cohort (*n* = 102) was partitioned once by stratified random sampling into a development set (DEV; *n* = 76) and an internal sealed split-sample hold-out dataset (*n* = 26; 13 per class). All feature selection, model search, and ranking steps were confined to DEV; the hold-out dataset was scored exactly once. Within DEV, the primary protocol was 5-fold stratified cross-validation repeated 10 times (50 folds), with feature selection refit inside each training fold to preclude leakage; scoring used balanced accuracy as the primary indicator, with macro-F1, accuracy, and ROC-AUC as secondary measures. Because the 50 training sets overlap, internal intervals use the Nadeau–Bengio corrected resampled variance, σ^2^(1/J + 1/(k − 1)), which widens the standard error approximately 3.8 fold, with subject-level stratified bootstrap (B = 2000) over out-of-fold predictions as a non-parametric check. Hold-out dataset intervals were exact or near-exact, computed with Clopper–Pearson analysis for accuracy and a Jeffreys Beta composite for balanced accuracy [40]. The rule for carrying a single pipeline through to internal scoring was fixed before the hold-out dataset was opened: the pipeline with the highest corrected lower confidence bound. Applying that rule to the development-set search over 270 candidate pipelines identified one model, which was then locked, refit on the full DEV set, and applied only once to the sealed subjects. 

**Figure 3:**
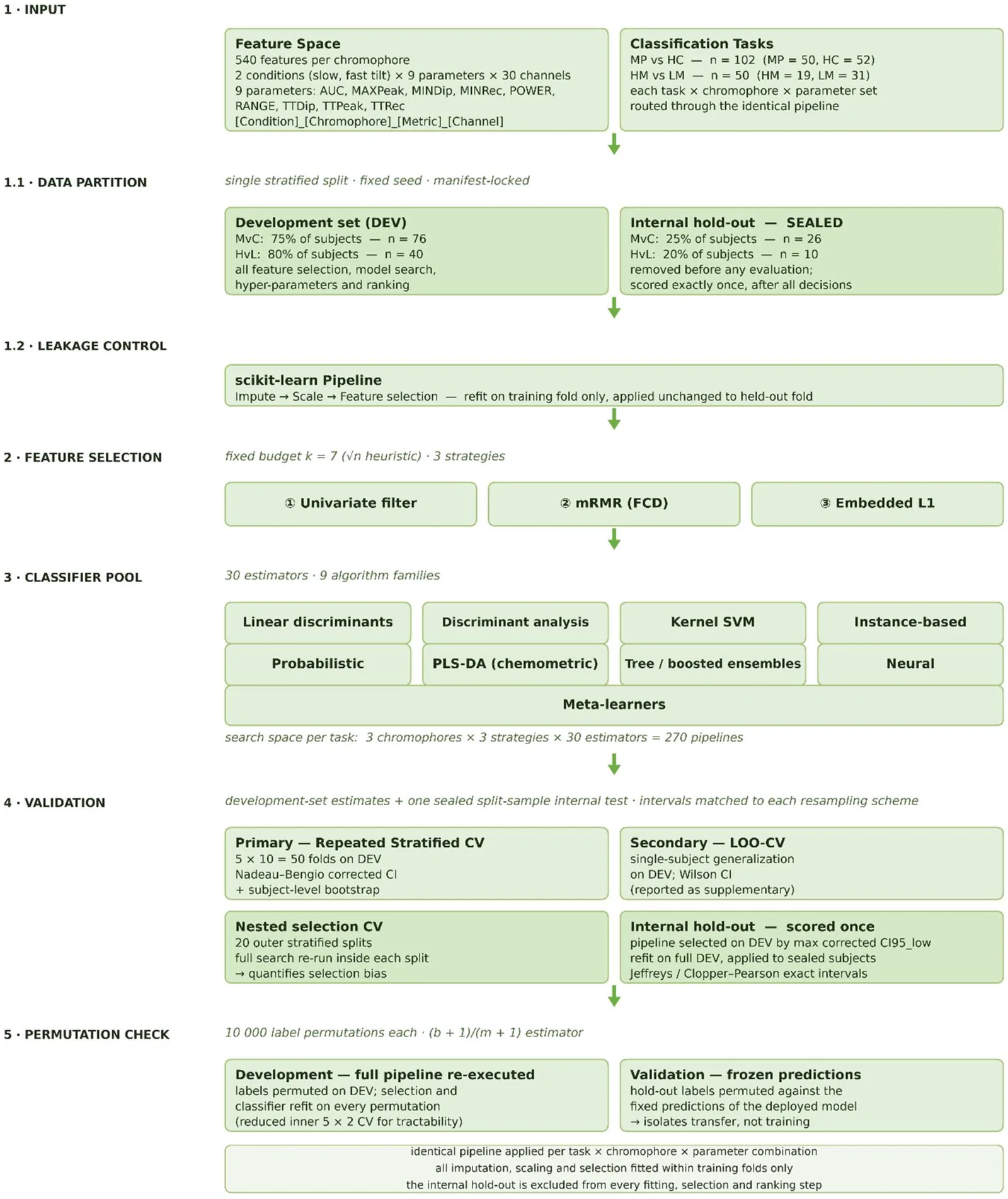
Schematic steps of classification pipeline.

Permutation check: Two label permutation tests were run, each addressing a different null. Internally, the 270 candidate pipelines (3 chromophores × 3 feature selection strategies × 30 classifiers) were re-executed on DEV under 10,000 random permutations of the class labels, testing whether the procedure extracts label-related structure beyond chance. A reduced inner scheme (RepeatedStratifiedKFold, 5 × 2) was used for tractability; the observed statistic under this scheme is marginally below the 5 × 10 estimate reported elsewhere. For validation, the hold-out dataset labels were permuted 10,000 times against the frozen predictions of the pre-selected development model, testing whether those predictions carry information about hold-out class membership [41] (Figure 3).

## 3. Results

### 3.1. Classification of Migraine Patients and Healthy Control Subjects (MP vs. HC, n = 102 (MP = 50, HC = 52))

**Figure 4:**
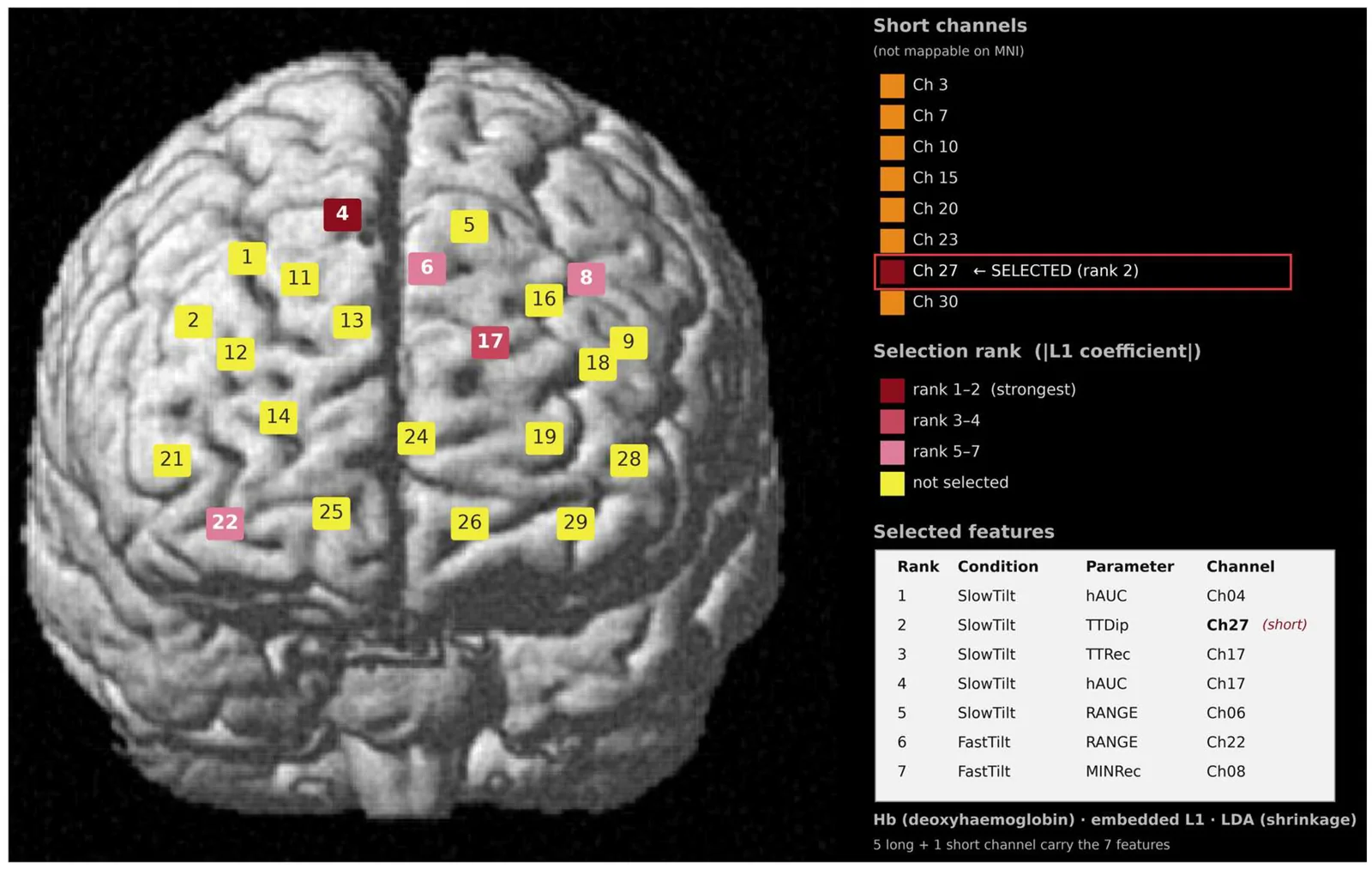
Channel locations of fNIRS probes mapped onto a standard brain template in MNI space. Channels are color coded by the rank of their contributing feature in the estimator selected on the development set (Hb, embedded L1 selection, and shrinkage LDA), ordered by the absolute magnitude of the L1 coefficient. Darker shades denote higher-ranked features (ranks 1–2, 3–4, and 5–7); yellow denotes channels not selected.

The best-performing classifier pipeline combined deoxyhemoglobin (Hb) parameters (hAUC, RANGE, TTDip, TTRec, and MINRec) selected by the embedded L1 (LASSO-penalized) feature selection method with shrinkage-regularized linear discriminant analysis. It reached 87% balanced accuracy in development-set cross-validation, 88% in repeated nested selection cross-validation, and 92% on the hold-out dataset when classifying the MP and HC groups (label permutation test *p* = 3 × 10^−4^). Five slow and two fast head-down-to-knees task features were among the top-ranked features (Figure 4).

### 3.2. High- vs. Low-Severity Migraine Patient Classification (HM vs. LM, n = 50 (HM = 19, LM = 31))

The best-performing classifier pipeline combined oxyhemoglobin (HbO) parameters selected by the embedded L1 (LASSO-penalized) feature selection method with linear support vector classification. It reached 65% balanced accuracy in development-set cross-validation, 45% in repeated nested selection cross-validation, and 62% on the hold-out dataset when classifying the HM and LM groups (label permutation test *p* = 0.44). Embedded L1 feature selection used a fixed penalty (C = 0.1); where fewer than *k* coefficients remained non-zero, the residual selections were tie-broken by column index and their rank order is not interpretable for this task.

## 4. Discussion

In the present study, we applied a broad, comprehensive range of machine learning classification algorithms to discriminate migraine patients from HC and high-severity migraine from low-severity subtypes, using fNIRS recordings acquired during a head-down-to-knees postural maneuver. Rather than classifying the raw signal directly, we derived a set of calculated hemodynamic parameters to sharpen the physiological interpretation of the response, and we used the prefrontal channels as the feature space without restricting the analysis to predefined regions of interest. Classification performance was strong for migraine vs. controls but below chance for migraine severity.

**Figure 5:**
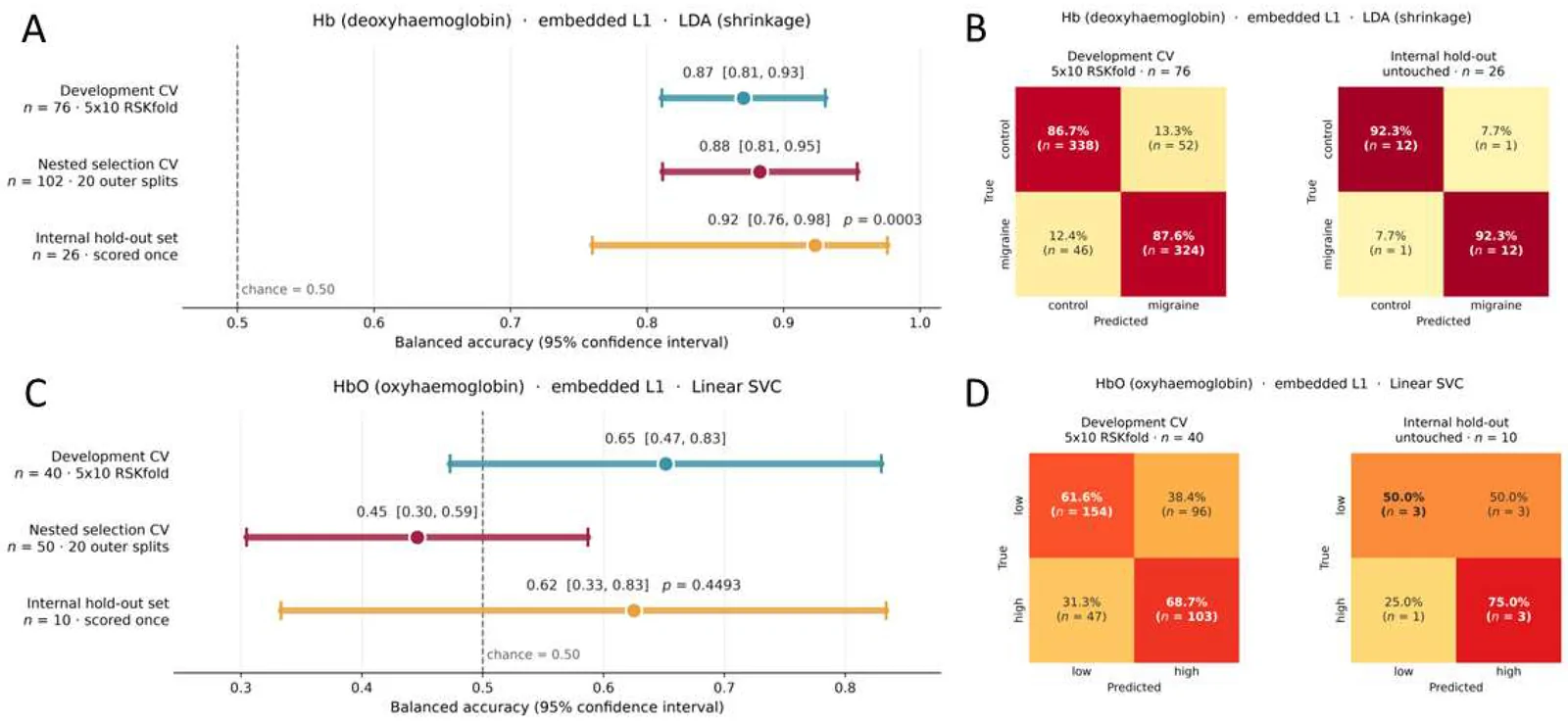
(**A**,**C**) Balanced accuracy with 95% confidence intervals at three levels of evidence, for the MP vs. HC task ((**A**); Hb, embedded L1 selection, and shrinkage LDA) and the HM vs. LM task ((**C**); HbO, embedded L1 selection, and linear SVC). (**B**,**D**) Confusion matrices for the same pipelines, row-normalized, for MP vs. HC (**B**) and HM vs. LM (**D**). Development-set matrices pool predictions from all 50 cross-validation folds, so each subject contributes one prediction per repeat ((**B**): 10 × 76 = 760 predictions; (**D**): 10 × 40 = 400); counts therefore exceed the number of subjects. Hold-out matrices report one prediction per hold-out subject ((**B**): *n* = 26; (**D**): *n* = 10) Color intensity is mapped automatically to the cell value, from white for the lowest counts to dark red for the highest. Shading is scaled for (**B**,**D**) panel together.

In the migraine patient vs. healthy control (MP vs. HC) task, the estimator selected on the development set (shrinkage-regularized linear discriminant analysis trained on L1 embedded deoxyhemoglobin (Hb) features) separated the two groups with 0.92 balanced accuracy on a sealed internal hold-out of 26 subjects never used for feature selection, model selection, or ranking (95% CI 0.76–0.98; sensitivity 0.92, specificity 0.92, precision 0.92, F1 0.92, AUC 0.99; permutation *p* = 3 × 10^−4^; Figure 5A,B). Sealed internal validation performance was also consistent with both development analyses: 0.87 (95% CI 0.81–0.93) under repeated stratified cross-validation of the development set, and 0.88 ± 0.06 (95% CI 0.81–0.95) under repeated nested selection cross-validation, in which the entire chromophore × feature selection strategy × classifier search was re-executed inside every outer split. Our work differs from prior fNIRS migraine classification studies; rather than a cognitive task, we used a vaso-mechanical challenge (postural head-down-to-knees maneuver) that alters cerebrovascular reactivity and autoregulation mechanisms directly. Despite this difference, our MP vs. HC classification performance is comparable. Chen et al. [17] separated migraine patients from controls using a QDA estimator on hemoglobin total (HbT) features from a mental arithmetic stress task, reporting 100% sensitivity and 75% specificity for CM, but that study included only four PFC channels and thirty-four subjects (13 HC, 9 CM, and 12 MOH). Kara Gulay et al. [18] also reported 92% balanced accuracy (89% sensitivity, 95% specificity) for a three-class MwA/MwoA/control task with XGBoost on time frequency HbO features of the left PFC during a Victoria Stroop test in a relatively small cohort (32 subjects: 8 MwA, 12 MwoA, and 12 HC). Erdoğan and Yükselen [42] achieved 85% accuracy (84% sensitivity, 95% specificity) in a four-class neuropsychiatric classification task that included interictal MwoA vs. controls, using an SVM estimator on oxyhemoglobin (HbO)-derived global efficiency metrics during a Stroop task in 33 subjects (13 HC, 20 migraine). Apart from the previous migraine classification studies, our cohort (102 subjects, 50 migraineurs, and 52 HC) is nearly three times the size. Our performance is also supported by the validation procedure: because the reported pipeline was chosen after inspecting cross-validated performance across 270 candidate pipelines, nested selection cross-validation was used to quantify the resulting optimism directly, performing a selection bias of only +0.03 balanced accuracy points. As repeated cross-validation folds share overlapping training sets, confidence intervals were computed with the Nadeau–Bengio corrected resampled variance rather than by treating folds as independent. The sealed, split-sample internal hold-out dataset was partitioned before any analysis, fixed in a persistent manifest and scored exactly once to confirm these estimates.

For the migraine vs. control classification, the highest-ranked features comprised two paramedian channels flanking the midline (Ch 04 and Ch 06) together with a single short separation channel (Figure 4). We interpret this as a composite hemodynamic signature, a joint readout of extracerebral, systemic vascular, and cortical responses to the postural maneuver rather than an isolated cortical signal. Long separation channels sample a mixed volume in which photons traverse scalp and skull and only a minority of the detected signal is of cortical origin, so superficial and cortical hemodynamics are inseparable without dedicated short separation regression [43]. That a short separation channel ranked among the top discriminating features indicates that extracerebral hemodynamics carry migraine-relevant information rather than constituting noise to be removed. Unlike cognitive tasks, postural maneuvers evoke prominent HbO and Hb changes through systemic autonomic activation [44], engaging systemic, autoregulatory, and cortical physiology concurrently by design. The paramedian position of Ch 04 and Ch 06 is anatomically compatible with a posture-loaded venous contribution. Both lie close to the course of the superior sagittal sinus and falx cerebri, such that their sensitivity volumes plausibly include a partial contribution from parasagittal drainage of cortical, dural, diploic, and emissary scalp veins. The head-down maneuver redistributes cerebral venous outflow from the internal jugular toward the vertebral venous system and raises intracranial pressure primarily through the hydrostatic cerebrospinal fluid column, which is the compartment that channels neighboring the midline are positioned to sample [45].

In the MP vs. HC classification task, the 92% accuracy was driven by a diverse calculated hemodynamic parameter set (hAUC, RANGE, TTDip, TTRec, and MINRec) on the Hb chromophore, indicating that the discriminative signal is distributed across many altered properties of the hemodynamic response rather than concentrated in a single parameter (Figure 4). Our multi-parameter Hb signal may be mechanistically informative. Systemic arterial and cardiac oscillations modulate arterial blood volume, which is primarily reflected by the HbO chromophore, whereas the venous-weighted Hb chromophore remains comparatively free of this systemic contamination. A multimodal fNIRS–fMRI study showed that task-evoked systemic artefacts appear in extracranial HbO recordings but are largely absent from Hb [46], and Hb has been identified as the chromophore most strongly coupled to the venous compartment that generates the BOLD signal [47]. Because our task is postural rather than cognitive, cerebrovascular components such as impaired vasoreactivity and altered autoregulation may be more evident in the migraine group, consistent with fNIRS studies that have highlighted such effects in Hb measurements [23,48]. Vascular involvement in migraine is independently supported by altered cerebrovascular reactivity and elevated arterial stiffness in a meta-analysis [49], blunted prefrontal reactivity to a vasomotor breath challenge [23], disrupted cerebral autoregulation on combined TCD–fNIRS assessment [48], and a high prevalence of orthostatic intolerance in migraine patients [50]. The high-accuracy separation of migraine achieved with a diverse hemodynamic parameter set also supports the view that migraine has a distributed, spectrum-level phenotype rather than a focal abnormality [51].

Another line of evidence comes from the classifier itself. The model carried forward for sealed validation testing was a shrinkage-regularized linear discriminant analysis, a classical linear classifier where the only added ingredient is a stabilizing adjustment to the way it estimates the relationships among features [52]. That adjustment matters when the number of subjects is modest relative to the number of features, because it keeps the classifier from chasing small fluctuations in the sample and holds its decision boundary steady. Among the leading candidates it varied least from one cross-validation fold to the next, and it was selected for sealed split-sample testing on that stability rather than on peak accuracy. A rejected HbO-based model looked stronger internally (0.89) but fell to 0.62 on the sealed hold-out dataset, whereas the stability-selected Hb model improved from 0.87 internally to 0.92 on the sealed hold-out dataset. That a simple, well-regularized linear classifier transferred so cleanly to unseen subjects reinforces the parameter-level finding: the migraine-related signal is not a subtle pattern requiring an elaborate model to detect, but a broad and consistent shift across several Hb response properties that a linear decision boundary can capture.

In the migraine severity task (high vs. low attack frequency, HM vs. LM), the estimator selected on the development set was a linear support vector classifier trained on L1 embedded oxyhemoglobin (HbO) features. Under repeated nested selection cross-validation balanced accuracy was 0.45 (95% CI 0.30–0.59), at or below the 0.50 chance level, and the sealed hold-out dataset gave 0.62 (95% CI 0.33–0.83; permutation *p* = 0.45) on ten subjects (Figure 5C,D). Development-set cross-validation returned an apparently higher 0.65, but its corrected lower bound (0.47) also fell below chance, and the mean best inner-fold score across nested splits was 0.69 while the corresponding outer split score was 0.45, giving a selection bias of +0.24 balanced accuracy points. We therefore report HM vs. LM classification as a negative result. With the present experimental cohort, postural task, and feature set, attack frequency severity was not separable above chance. Class imbalance (19 HM vs. 31 LM) and the small development-set sample size are the most immediate explanations, but the below-chance level of performance requires further consideration. 

Prophylactic medication is a plausible major confounder acting in the direction that suppresses separability of migraine severity. Propranolol prophylaxis significantly reduces cerebrovascular reactivity to functional activation in migraineurs, and flunarizine has been reported to normalize previously elevated reactivity [53]. Since higher attack frequency is one of the principal clinical indications for initiating prophylaxis, the more severely affected group is also the group whose vaso-mechanical and autoregulatory responses are most likely to be pharmacologically blunted. Any severity-graded hemodynamic difference would then be compressed toward the low-attack-frequency group’s profile, flattening the contrast the classifier is asked to detect. This confound may be structurally bound to our grouping variable. The HM and LM groups did not differ significantly in disease duration, so the contrast rests on current attack frequency and on MOH status, which separated the groups completely (Table 1). This co-separation is expected since increased attack frequency can be the reason for medication overuse. Eight of the nineteen HM patients had MOH, and their prefrontal hemodynamic profiles may differ from those of the remaining eleven without it. MOH has also been found to be associated with hypometabolism of the orbitofrontal cortex that persists even after successful medication withdrawal [54]. Duration and frequency dissociation is expected as progression from low-frequency to high-frequency or chronic migraine occurs in only a minority of patients, and the established risk factors are largely comorbidity-related and modifiable rather than a function of time since the disease onset [55]. Attack frequency thus indexes current migraine activity rather than cumulative burden, and the variable defining our groups is therefore the one most likely to have been pharmacologically modified.

Additionally, the feature space may not encode the property that distinguishes migraine severity. Our descriptors are scalar summaries of response magnitude and latency (hAUC, MAXPeak, MINDip, RANGE, POWER, TTPeak, TTDip, and TTRec), which characterize the amplitude and timing of a single evoked hemodynamic response but not its higher-order temporal structure. Bäcker et al. [56] found that the absence of habituation of the cerebral blood flow velocity response was significantly more pronounced in migraine patients with high attack frequency (≥4/month) than in those with low frequency (<4/month). However, our scalar amplitude and latency descriptors are largely blind to habituation slope, response variability, and time frequency content. Kara Gulay et al. [18] also achieved 92% balanced accuracy for a three-class migraine with aura/migraine without aura/control problem using time frequency HbO features rather than scalar amplitude metrics, although the presence of aura is a categorical clinical phenotype rather than a graded severity dimension. That the same scalar hemodynamic parameters supported robust MP vs. HC discrimination (0.92 on the sealed hold-out) while failing entirely for HM vs. LM suggests that these descriptors are adequate for a categorical presence or absence contrast but insufficient for a graded one.

### 4.1. Limitations and Future Directions

Several limitations constrain the present findings, particularly for the severity contrast, which we could not separate above chance. Medication status was not entered into the classification tasks as a covariate or stratification factor for the healthy control, migraine, or severity subtype groups. Because higher attack frequency is itself the principal indication for starting prophylaxis, the more severely affected patients are also those whose vaso-mechanical responses are most likely to be pharmacologically blunted. Future prospective studies should record agent, dose, duration, and washout in a standardized manner across multiple visits and pre-specify medication-stratified subgroup analyses, including medication overuse status.

Our sample does not support subgroup-level classification. Clinical variables including aura status, headache severity scales, and prophylaxis history, as well as participant characteristics including sex, body mass index, mean arterial pressure, habitual exercise, caffeine intake, and depression and anxiety scores were recorded but were not entered into the classification tasks (Table S1). The present 19/31 imbalance between the HM and LM groups, together with the small group sizes, would make stratification on any known modifier of the cerebrovascular response prone to overfitting and could reduce cell sizes to an uninterpretable level [57]. Larger cohorts, recruited prospectively with respect to these variables and with a more sex-balanced distribution, are recommended for further studies. Patients with autonomic dysregulation disorders or non-migraine chronic pain could be included to distinguish the effects of migraine pain more precisely.

The postural maneuver was not instrumented during our experiment. Angular velocity and peak flexion angle were not quantified, so between-subject variation in how the head-down maneuver was executed remains an uncontrolled source of hemodynamic variance. Concurrent kinematic recording, such as a head-mounted inertial measurement unit, an fNIRS system with an integrated accelerometer, or video-based motion capture would allow trial-by-trial normalization of the postural head-down maneuver task.

Finally, our hemodynamic feature space may be too limited for graded contrasts. Scalar measures of response magnitude and latency were sufficient to distinguish MP from HC categorically, but not to classify migraine severity, where the reported differences relate more to response dynamics than to amplitude alone. Richer representations, including habituation slopes across repeated maneuvers, wavelet or time frequency decompositions, and connectivity or entropy measures, may be needed to resolve migraine severity. 

### 4.2. Conclusions

The presented work demonstrates the utility of monitoring cerebral hemodynamics with fNIRS during a brief postural head-down-to-knees maneuver, performed without a tilt table, for separating interictal migraine patients from controls screened for the absence of migraine, serious chronic conditions, and hypertension. Our methodology could distinguish patients with migraine from controls with 0.92 balanced accuracy on a sealed internal hold-out dataset that was scored exactly once. This performance was achieved by a hypothesis-free pipeline that searched three chromophores, nine hemodynamic parameters, and all thirty channels across three feature selection strategies and thirty classifiers, without a predefined region of interest or privileged chromophore. The discriminative features originated from deoxyhemoglobin and were distributed across amplitude and latency descriptors and weighed towards paramedian and short separation channels, consistent with a venous and systemically loaded response to a hydrostatic challenge rather than with focal cortical activation. By contrast, classification based on attack frequency severity did not exceed chance under nested validation and is reported as a negative result. A migraine severity subcohort of 50 patients, larger than the full sample of any prior fNIRS migraine classification study, was still insufficient to resolve a graded contrast. In summary, fNIRS hemodynamic parameters derived from postural tasks offer a high-performance categorical separation of interictal migraine from controls. Establishing diagnostic specificity will require prospective comparison against other primary headache disorders, non-migraine chronic pain, and autonomic conditions, and generalizability across sex, medication status, aura, and headache severity should be assessed in larger cohorts.

## Figures and Tables

**Table 1:** Clinical characteristics and prophylactic medication by migraine group.

Variable	LM (*n* = 31)	HM (*n* = 19)	n	Test	Statistic	*p*	Effect Size
Age (years)	35.0 ± 10.9	33.9 ± 12.6	50	Welch t	t(34.0) = 0.31	0.759	g = 0.09 [−0.49, 0.68]
Migraine duration (years)	7.9 ± 7.6	7.3 ± 8.8	50	Welch t	t(33.9) = 0.25	0.807	g = 0.07 [−0.51, 0.66]
Headache intensity (VAS 0-10)	8.4 ± 1.7	8.2 ± 1.5	49	Welch t	t(41.9) = 0.56	0.579	g = 0.16 [−0.43, 0.75]
HIT-6 score	63.4 ± 6.2	66.2 ± 6.5	50	Welch t	t(36.9) = −1.47	0.150	g = −0.43 [−1.02, 0.17]
Sex (% male)	5 (16.1%)	0 (0.0%)	50	Fisher	chi2(1) = 1.85	0.142	OR = 0.12 [0.01, 2.37]
Aura (% yes)	5 (16.1%)	1 (5.3%)	50	Fisher	chi2(1) = 0.49	0.387	OR = 0.29 [0.03, 2.69]
MOH (% yes, 15 or more days with acute medication)	0 (0.0%)	8 (42.1%)	50	Fisher	chi2(1) = 12.56	<0.001 *	OR = 46.57 [2.48, 872.99]
Attack frequency (per month)	2.7 ± 1.7	8.3 ± 4.6	50	Welch t	t(21.1) = −5.06	<0.001 *	g = −1.75 [−2.43, −1.07]
Prophylactic agent class:							
Beta blocker	5 (16.1%)	2 (10.5%)	50	-	-	-	Descriptive
Antidepressant	1 (3.2%)	6 (31.6%)	50	-	-	-	Descriptive
Botulinum toxin	1 (3.2%)	3 (15.8%)	50	-	-	-	Descriptive
Nerve block (GON)	2 (6.5%)	3 (15.8%)	50	-	-	-	Descriptive
Antiepileptic	2 (6.5%)	2 (10.5%)	50	-	-	-	Descriptive
Calcium channel blocker	0 (0.0%)	1 (5.3%)	50	-	-	-	Descriptive
Anti-CGRP monoclonal antibody	0 (0.0%)	0 (0.0%)	50	-	-	-	Descriptive
Acute attack agent class:							
Triptan	5 (16.1%)	1 (5.3%)	50	-	-	-	Descriptive
Ergot/NSAID	1 (3.2%)	0 (0.0%)	50	-	-	-	Descriptive

Continuous variables: mean ± SD. Categorical variables: n (%). OR = odds ratio, HM relative to LM. Patients receiving more than one agent are counted in each class; counts exceed the number of patients treated. * *p* < 0.001.

**Table 2:** List of hemodynamic parameters with formula and definitions.

Parameter	Formula (C ∈ {HbO, Hb, HbT})	Definition
hAUC	$AUC\left(C\right)=\sum_{n=1}^{N}C\left(n\right)$	Total area under signal
MAXPeak	MAXPeak(C) = max C(n), n∈TS	First peak maximum value
MINDip	MINDip(C) = (C(n)), $n\in TS_{Dip}$	Initial dip minimum value
MINRec	MINRec(C) = (C(n)), $n\in TS_{Rec}$	Recovery-phase minimum value
POWER	$POWER\left(C\right)=\frac{1}{N}\sum_{n=1}^{N}C{\left(n\right)}^{2}$	Power of signal
RANGE	RANGE(C) =(C(n)) −(C(n)) n∈TS	Maximum to minimum amplitude difference
TTDip	TTDip(C) = argarg (C(n)) , $n\in TS_{Dip}$	Latency to initial dip
TTPeak	TTPeak(C) = argarg (C(n)) , n∈TS	Latency to first peak
TTRec	TTRec(C) = argarg (C(n)) , $n\in TS_{Rec}$	Latency to recovery minimum

Note: C(t) denotes the chromophore signal at time t, where C ∈ {HbO (oxyhemoglobin), Hb (deoxyhemoglobin), HbT (total hemoglobin)}. TS denotes the full epoch window; $TS_{Dip}$ and $TS_{Rec}$ denote the dip and recovery sub-windows, respectively.

## Data Availability

Relevant data of the study are provided as Supplementary Material.

## Supplementary Materials

The following supporting information can be downloaded at: https://www.mdpi.com/article/10.3390/brainsci16090927/s1. Table S1: Cohort characteristics; File S1: All hemodynamic analysis code and classification code.

## Abbreviations

The following abbreviations are used in this manuscript

au	Arbitrary unit
BOLD	Blood-oxygen-level-dependent signal
CGRP	Calcitonin gene-related peptide
CI95_low/CI95_high	Lower and upper bounds of the 95% confidence interval
CM	Chronic migraine
CV	Coefficient of variation
DEV	Development set used for feature selection, model search, and ranking
EM	Episodic migraine
embedded_l1_top7	Embedded feature selection via L1 (Lasso) regularization; top seven features retained
F1 macro	Macro-averaged F1 score across all classes
fNIRS	Functional near-infrared spectroscopy
FS	Feature selection
GON	Greater occipital nerve (block)
hAUC	Hemodynamic area under the curve; signed integral of the chromophore signal over the epoch
Hb	Deoxyhemoglobin concentration change (μmol/L)
HbO	Oxyhemoglobin concentration change (μmol/L)
HbT	Total hemoglobin concentration change (μmol/L)
HC	Healthy control
HFEM	High-frequency episodic migraine
HIT-6	Headache Impact Test-6
HM	High-severity migraine class (HFEM + CM)
ICHD-3	International Classification of Headache Disorders, 3rd edition
LDA	Linear discriminant analysis
LDA_shrinkage	Linear discriminant analysis with Ledoit–Wolf shrinkage covariance estimator
LFEM (LM)	Low-frequency episodic migraine (low-severity migraine class)
LogReg_EN_bal	Logistic regression with elastic net regularization and class-balanced sample weighting
LogReg_EN_lightL1	Logistic regression with elastic net regularization and reduced L1 penalty ratio
LogReg_L1	Logistic regression with L1 (Lasso) regularization
MAXPeak	Maximum signal value within the peak sub-window
MINDip	Minimum signal value within the initial-dip sub-window
MINRec	Minimum signal value within the recovery sub-window
ML	Machine learning
MLP	Multilayer perceptron
MNI	Montreal Neurological Institute (coordinate space)
MOH	Medication overuse headache
MP	Migraine patient
mRMR	Minimum redundancy maximum relevance
mrmr_top7	Filter-based feature selection via the minimum redundancy maximum relevance criterion; top seven features retained
MwA/MwoA	Migraine with aura/migraine without aura
OD	Optical density
PFC	Prefrontal cortex
PLS-DA	Partial least squares discriminant analysis
POWER	Mean squared amplitude of the signal over the epoch
QDA	Quadratic discriminant analysis
RANGE	Global peak-to-peak amplitude of the signal over the epoch
ROC AUC	Area under the receiver operating characteristic curve
RSK	Repeated stratified K-fold cross-validation
std	Standard deviation of balanced accuracy across folds
SVC_RBF	Support vector classifier with radial basis function kernel
SVM/SVC	Support vector machine/support vector classifier
TCD	Transcranial Doppler
TS	Full epoch window used for hemodynamic feature extraction
TTDip	Latency from epoch onset to MINDip
TTPeak	Latency from epoch onset to MAXPeak
TTRec	Latency from epoch onset to MINRec
